# Work Stress and Alcohol Use

**Published:** 1999

**Authors:** Michael R. Frone

**Affiliations:** Michael R. Frone, Ph.D., is a senior research scientist at the Research Institute on Addictions, State University of New York at Buffalo, in Buffalo, New York

**Keywords:** employee, work related factor predisposing to AODU (AOD [alcohol or other drug] use, abuse, and dependence), alienation, psychological stress, workplace context, theoretical model, problematic AOD use, social role, literature review

## Abstract

Employees who drink heavily or who abuse or are dependent on alcohol can undermine a workforce’s overall health and productivity. To better understand the reasons behind employee abusive drinking and to develop more effective ways of preventing problem drinking in the workforce, researchers have developed a number of paradigms that guide their research. One such paradigm is the alienation/stress paradigm, which suggests that employee alcohol use may be a direct or indirect response to physical and psychosocial qualities of the work environment. Although in the alcohol literature, work alienation and work stress traditionally have been treated as separate paradigms, compelling reasons support subsuming the work-alienation paradigm under a general work-stress paradigm. Researchers have developed several models to explain the relationship between work stress and alcohol consumption: the simple cause-effect model, the mediation model, the moderation model, and the moderated mediation model. Of these, the moderated mediation model particularly stands out, because it simultaneously addresses the two fundamental issues of how and when work stressors are related to alcohol use. Recent research supports a relation of work-related stressors to elevated alcohol consumption and problem drinking. Future research should focus on the relation between work stressors and alcohol use among adolescents and young adults, because they are just entering the workforce and are the most likely to engage in heavy drinking. Longitudinal studies also are needed to better explain the relation between work stress and alcohol use.

Employee alcohol use^1^—whether or not it occurs on the job—is an important social policy issue, because it can undermine employee health as well as productivity. From a managerial perspective, the specific problems created by alcohol or other drug (AOD) use may include impaired performance of job-related tasks, accidents or injuries, poor attendance, high employee turnover, and increased health care costs (e.g., Ames et al. 1997; Dawson 1994; Frone 1998; Martin et al. 1994; Normand et al. 1994; Roman and Blum 1995). These outcomes may reduce productivity, increase the costs of doing business and, more generally, impede employers’ ability to compete effectively in an increasingly competitive economic environment. It is therefore not surprising that alcohol researchers, as well as researchers in the management and economics fields, take considerable interest in the factors that cause or explain employee alcohol use.

The literature on the causes of employee alcohol use generally takes one of two perspectives. The first perspective views the causes of employee alcohol use as external to the workplace. In other words, an employee may have a family history of alcohol abuse that leaves him or her vulnerable to developing drinking problems, have personality traits reflecting low behavioral self-control that make it difficult to avoid alcohol, or experience social norms and social networks outside work—such as friends who drink heavily—that affect drinking behavior (e.g., Ames and Janes 1992; Normand et al. 1994; Trice and Sonnenstuhl 1990).

Although external factors clearly influence employee drinking habits, a second perspective views the causes of employee alcohol use as arising, at least in part, from the work environment itself. This perspective can be further disaggregated into several narrower paradigms. Although researchers differ somewhat in how they label and categorize those narrower paradigms (for reviews, see Ames and Janes [1992] and Trice and Sonnenstuhl [1990]), three versions appear consistently in the literature:

- The *social control* paradigm suggests that alcohol use may be higher among employees who are not integrated into or regulated by the work organization. Thus, two important risk factors in the social control paradigm are low levels of supervision and low visibility of work behavior (Trice and Sonnenstuhl 1990).
- The *culture/availability* paradigm suggests that work settings where alcohol is physically or socially available may promote alcohol use among employees (Ames and Grube 1999; Ames and Janes 1992; Trice and Sonnenstuhl 1990).
- Physical availability of alcohol at work is defined as the ease with which alcohol can be obtained for consumption on the job, during breaks, and at work-related events (Ames and Grube 1999). Social availability of alcohol at work is defined as the degree to which fellow workers support drinking either off or on the job (Ames and Grube 1999; Ames and Janes 1992; Trice and Sonnenstuhl 1990).
- The *alienation/stress* paradigm suggests that employee alcohol use may be a response to the physical and psychosocial qualities of the work environment (Ames and Janes 1992; Trice and Sonnenstuhl 1990), such as work demands on an employee, an employee’s level of boredom, lack of participation in decisionmaking, and interpersonal conflict with supervisors and coworkers.

The remainder of this article focuses on three issues. First, it describes the alienation/stress paradigm in more detail. Second, the article reviews recent research testing the alienation/stress paradigm, focusing on research conducted during the 1990s; this body of research has yet to be reviewed and has become broader and more sophisticated than earlier research testing the alienation/stress paradigm. (For reviews of earlier research conducted during the 1980s, see Cooper et al. [1990], Martin [1990], and Trice and Sonnenstuhl [1990]). Finally, the article makes several suggestions for focusing and strengthening future research.

## Work-Stress Paradigm

In the alcohol literature, work alienation and work stress typically have been treated as separate paradigms (e.g., Trice and Sonnenstuhl 1990). The work-alienation paradigm focuses on work characteristics that lead to unenriched jobs, such as those in which workers use only minimal skills, have little job control (e.g., lack control over the pace of work or its content), and have little or no input into decisionmaking. In contrast, the work-stress paradigm emphasizes other potentially aversive work conditions, which are labeled “work stres-sors.” Common work stressors include dangerous work conditions; noxious physical work environments (e.g., conditions that are too hot or cold, noisy, or dirty); interpersonal conflict with supervisors or coworkers; heavy workloads; unfair treatment regarding pay, benefits, and promotions; and job insecurity (e.g., threat of layoffs). Trice and Sonnenstuhl (1990) argued that the stress and alienation paradigms are conceptually distinct, because the alienation paradigm assumes that work is universally important in people’s lives, whereas the stress paradigm does not make this assumption. In other words, the alienation paradigm proposes that factors leading to unenriched jobs will be aversive to all employees, whereas the stress paradigm suggests that work stressors may not be aversive to all employees, because work is not universally important. Although Martin (1990) pointed out many similarities between the alienation and stress paradigms, he maintained a distinction between them in his review of the literature.

Four compelling reasons, however, support subsuming the work-alienation paradigm under a general work-stress paradigm:

1. The literature on work stress includes workplace alienation factors in taxonomies of work stressors and in major models of work stress (for a review, see Hurrell et al. 1998).
2. Both paradigms are based on the assumption that alcohol use represents a means of regulating negative emotions (e.g., depression, anxiety, or anger) or thoughts that result from aversive work environments.
3. Despite a basic assumption of the work-alienation paradigm, evidence shows that work does not have a high level of importance in every person’s life. Variability in the psychological importance of work exists from person to person (Frone et al. 1997*a*).
4. Both theoretical and empirical research suggest that individual differences in the psychological importance of work may be important in explaining when work stressors will be related to alcohol use (e.g., Frone et al. 1997*a*).

Based on these considerations, this article simply treats work-alienation factors as work stressors.

Even if one subsumes the alienation paradigm into a broad work-stress paradigm, the focus of past work-stress research has been restrictive in that attention has generally focused on stressors that occur *within* the work role (e.g., work demands and conflict with coworkers). Another type of work-related stres-sor, however, occurs when the demands of work begin to interfere with other social roles. For example, work-family conflict represents the extent to which work and family life interfere with one another (Frone et al. 1997*c*). This type of stressor should be incorporated into the work-stress paradigm, because only employed people can experience it. This article, however, separately examines past research on work stressors (within-role stressors) and work-family conflict (between-role stressors) because they represent qualitatively different aspects of a person’s work life.

## Evidence From Research

A comprehensive review of the entire body of literature on work stressors and alcohol use is beyond the scope of this article^2^; it does, however, offer a taxonomy consisting of four work-stress models that provides a useful way of organizing recent research. The following sections define each model and summarize *representative* studies. Although a few studies explicitly tested more than one model, the primary goal of most studies was to test one of the four models (see figure).

Among the studies reviewed in this article, two basic research designs are used. The most common research design is the cross-sectional study, in which work stressors and alcohol outcomes are measured at the same time. Although the underlying hypothesis tested in these studies is that work stressors cause alcohol use, cross-sectional studies *cannot* support conclusions regarding cause and effect. Those studies can only document that work stressors are related to alcohol use. A cross-sectional relation may be attributable to the fact that work stressors cause alcohol use. However, equally plausible is the concept that alcohol use may cause increased levels of work stress or that the relation is spurious, because some other unmeasured variables, such as personality traits, cause some people to choose stressful jobs and to drink heavily. The second research design is the longitudinal study, in which work stressors and alcohol outcomes are measured at two or more different points in time. In the typical longitudinal study, work stressors assessed at baseline (e.g., 1996) are used to predict alcohol use at a later point in time (e.g., 1997) after controlling for initial differences in alcohol use at baseline. Although less common, longitudinal studies offer more convincing evidence that exposure to work stressors causes increases in alcohol use. Unless a study is explicitly labeled as longitudinal, the reader should assume that the studies reviewed below are cross-sectional.

### Simple Cause-Effect Model

The first model presented in the figure is the simple cause-effect model of work stress and alcohol use. Research based on this model simply attempts to document an overall relation between various work stressors and different dimensions of alcohol use, usually controlling for basic demographic variables, such as age, gender, income, and occupation. Support for the simple cause-effect model is mixed. For example, Parker and Farmer (1990) and Roxburgh (1998) reported that low levels of job complexity (i.e., jobs that require little thought and independent judgment) are related to impaired control over drinking and elevated daily consumption. Ragland and colleagues (1995) found that a measure of work problems was positively related to heavy drinking^3^ and average weekly consumption; job demands and job control, however, were not related to alcohol use. Using longitudinal data, Crum and colleagues (1995) reported that men holding jobs that were high in demands and low in job control were more likely to develop either an alcohol abuse or alcohol dependence disorder than were men in jobs that lacked one or both of these two job stressors. The researchers, however, found no such relation among women. Hemmingsson and Lundberg (1998) found that low job control, but not high job demands, was associated with a diagnosis of alcohol abuse or dependence among men. These researchers did not include women in their study.

The studies summarized so far suggest that jobs low in complexity and control and high in demands are related to increased employee alcohol use. Some evidence indicates that these work stressors may be more strongly related to alcohol use among men. Nonetheless, a number of studies assessing similar work stressors have failed to support the simple cause-effect model (Frone et al. 1997*a*; Greenberg and Grunberg 1995). Furthermore, even when gender differences are found in the strength of the relation between work stressors and alcohol use, no clear pattern exists across studies (Romelsjo et al. 1992; Roxburgh 1998).

The inconsistent findings from studies testing the simple cause-effect model are not surprising, because the model has two inherent limitations. First, the model is based on the premise that work stressors are causal antecedents of alcohol use for all, or at least many, employees. Although most adults consume alcohol, it is unlikely that most adults use alcohol to cope with unpleasant work conditions. Many coping behaviors, such as talking to friends or relatives, exercise, leisure activities, and addressing work problems at their source, relieve the resulting negative emotions from work stressors more effectively and have fewer negative side effects than alcohol consumption. It may be more reasonable to assume that only employees who lack certain resources or who have certain vulnerabilities (e.g., holding the belief that alcohol use relieves negative emotions or having heavily drinking peers) will use alcohol to cope with work stressors. If this assumption is true, then researchers who do not identify subgroups at risk for stress-induced drinking may have inconsistent and nonsignificant findings.

The second limitation is that even if the simple cause-effect model supports a relation between work stressors and alcohol use, no information is provided about why work stressors cause increased alcohol use. That is, the model makes no attempt to account for intervening variables, such as negative emotions, that would explain how work stressors are related to alcohol use. The underlying assumption of the simple cause-effect model of work stress and alcohol use is that work stressors cause negative emotions, which, in turn, cause alcohol use to relieve those emotions. Nonetheless, this assumption needs to be tested; failing to model intervening variables may render a study less likely to find a work stressor–alcohol relation. These observations have motivated many researchers to move beyond simple models of work stress and alcohol use (Wilsnack and Wilsnack 1992), as described in the next sections.

### Mediation Model

The mediation model explicitly incorporates the variables thought to link work stressors to alcohol use, such as sadness or anger (i.e., negative affect), inability to relax, and the drinker’s reason for drinking (i.e., drinking motives), such as to “ let off steam.” By including these mediating (i.e., intervening) variables, the mediation model goes beyond the simple cause-effect model by trying to explain *why* or *by what mechanism* work stressors are related to alcohol use.

**Figure:**
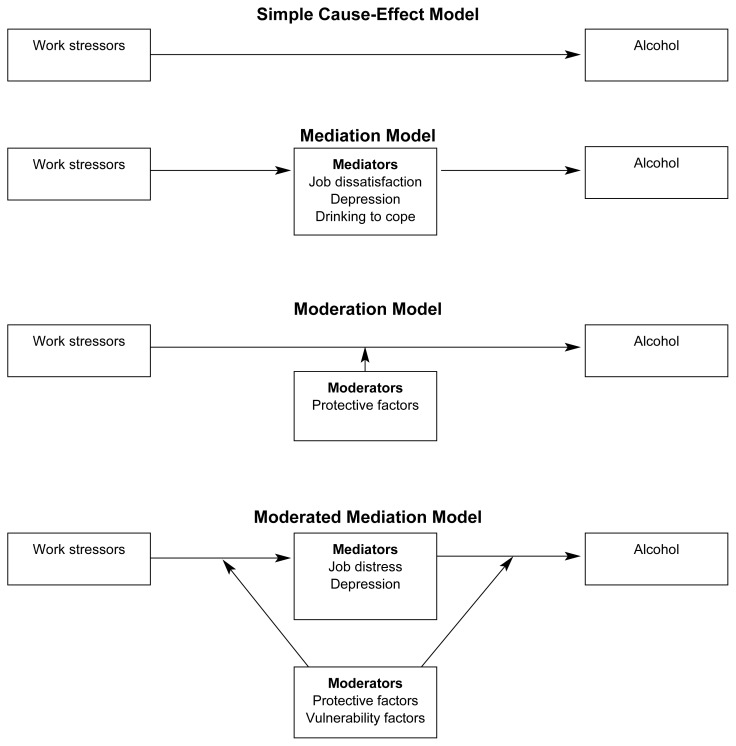


Although two studies (Cooper et al. 1990; Kawakami et al. 1993) failed to support the mediating role of negative affect, a number of studies support mediated models of work stress. For example, Vasse and colleagues (1998) reported that high work demands and poor interpersonal relations with supervisors and coworkers were positively related to anxiety, which was positively related to average weekly alcohol consumption. Martin and colleagues (1996) found that job demands and low job control were related to higher levels of drinking to cope with negative affect, which was positively related to both average monthly alcohol consumption and problem drinking. In addition, one study examined the mediating role of both job dissatisfaction and drinking to cope. Greenberg and Grunberg (1995) reported that workers who felt their skills were underused, had low job control, and had little participation in deci-sionmaking were more likely to be dissatisfied with their jobs. This dissatisfaction was, in turn, positively related to drinking to cope, which was positively related to both heavy drinking and problem drinking.

### Moderation Model

The moderation model explicitly includes variables that moderate the relation between work stressors and alcohol use. This model is an interactional one, in which work stressors interact with certain variables that either place a worker at increased risk for or protect the worker from developing problems with alcohol. The basic premise is that the strength of the relation between work stressors and alcohol use differs as a function of the level of the risk and protective variables. The moderation model, therefore, goes beyond the simple cause-effect model by trying to explain *when* or *under what conditions* work stressors are related to alcohol use. In other words, work stressors are not assumed to be related to alcohol use among all employees.

Several studies have tested this model. For example, building from identity theory,^4^ Frone and colleagues (1997*a*) showed that both job demands and the lack of a clearly defined role at the workplace (i.e., role ambiguity) were positively related to heavy drinking only among employees who reported that their work role was psychologically important for self-definition. Among participants who reported low psychological importance of work, the work stressors were unrelated to heavy drinking. Grunberg and colleagues (1999) reported that work pressure predicted higher average daily alcohol consumption and problem drinking among people who reported that they typically drank to relax and forget about problems than among people who did not drink for those reasons. Among people in the latter group, work pressure was unrelated to the alcohol outcomes. Finally, Parker and Harford (1992) examined the moderating influence of gender-role attitudes on the relation of job competition to alcohol use. Traditional gender-role attitudes represent the belief that men should be breadwinners and women should take care of home and family. Egalitarian gender-role attitudes represent the belief that men and women should share breadwinning and domestic responsibilities. For women, job competition was more strongly related to drinks per drinking occasion, whereas loss of control over drinking was more strongly related among those with more traditional gender-role attitudes. In contrast, for men, job competition was more strongly related to drinks per drinking occasion and loss of control over drinking among those individuals with more egalitarian gender-role attitudes.

### Moderated Mediation Model

This model combines the features of the mediation and the moderation models. By explicitly including both mediating and moderating variables, the moderated mediation model goes beyond each of the other three models by simultaneously trying to explain *how* as well as *when* work stressors are related to alcohol use. Several variations of this model can be devised, depending on the moderator variables. For example, one could have a model in which a given vulnerability or protective factor only moderates one of the paths connecting work stressors to alcohol use. One also might hypothesize moderating effects on both paths, but with different vulnerability or protective factors moderating each path.

Only two studies have proposed and tested a moderated mediation model of work stress and alcohol use. Building from several different theoretical frameworks, Cooper and colleagues (1990) and Grunberg and colleagues (1998) hypothesized that (1) work stressors are positively related to job dissatisfaction and (2) job dissatisfaction is positively related to alcohol use among vulnerable people. Supporting the first hypothesis, Cooper and colleagues (1990) found that work demands and lack of job control were positively related to job dissatisfaction. Likewise, Grunberg and colleagues (1998) reported that high levels of job demands, interpersonal criticism from supervisors and coworkers, and feeling stuck in one’s job were positively related to job dissatisfaction. With regard to the second hypothesis, Cooper and colleagues (1990) found some support that positive alcohol expectancies,^5^ the general belief that one is competent, and coping with difficult situations by avoiding them (i.e., avoidance coping) had a moderating influence. For example, job dissatisfaction was more strongly related to problem drinking among people who reported high levels of avoidance coping. Likewise, Grunberg and colleagues (1998) reported that job dissatisfaction was related to problem drinking among those who reported that they drank to reduce negative emotions. Among people who did not drink for this reason, job dissatisfaction was unrelated to problem drinking.

## Work-Family Conflict

As discussed earlier, work-family conflict represents the extent to which demands and responsibilities in one role (work or home) interfere with meeting the demands and responsibilities in the other role (home or work) (Frone et al. 1997*c*). Because work-family conflict involves difficulties with integrating work and family life, it is a between-role stressor that may cause increased alcohol consumption. In a review of workplace predictors of women’s drinking, Shore (1992) concluded that conflict between work and other social roles is not predictive of alcohol use. This conclusion was based on the finding that women who had a large number of social roles (e.g., employee, spouse, parent, and church member) did not report higher levels of alcohol consumption or problem drinking than did women who had only a few social roles. This research, however, suffers from an important conceptual limitation. The number of social roles a person holds is not a good indicator of the amount of conflict among those roles. Having several social roles is a necessary but not sufficient condition for inter-role conflict. Depending on a variety of circumstances, some people with work and family roles experience no conflict between the roles, whereas other people experience a high degree of conflict between them (Frone et al. 1997*c*). Alcohol researchers in this area, therefore, need to measure work-family conflict directly. Several recent studies have done so and have provided tests of the first three models shown in the figure.

### Simple Cause-Effect Model

One study, which used a small sample of 71 workers, failed to find a relation between work-family conflict and the amount of alcohol consumed over the preceding 7 days (Steptoe et al. 1998). In contrast, Bromet and colleagues (1990) reported that work-family conflict was positively related to daily alcohol consumption in a sample of blue-collar working women. Frone and colleagues (1996) found that work-family conflict was positively related to heavy drinking among men and women in two community samples of employed parents. In a longitudinal followup study, Frone and colleagues (1997*b*) reported that work-family conflict assessed in 1989 predicted heavy drinking in 1993 among men and women. Using a representative national sample, Frone (in press) reported that work-family conflict was positively related to a diagnosis of AOD dependence, but not AOD abuse, among men and women. In summary, past research based on the simple cause-effect model provides consistent evidence that work-family conflict is related to elevated alcohol use among men and women. Nonetheless, this model does not explain why work-family conflict is related to alcohol use or whether certain people are more prone to alcohol use when exposed to work-family conflict. These two issues are addressed in the next sections.

### Mediation Model

Two studies tested the process that explains why work-family conflict is related to alcohol use. Both studies tested the general hypothesis that work-family conflict causes negative emotions, which in turn cause increased alcohol use. Vasse and colleagues (1998) found that work-family conflict was positively related to overall emotional distress, which in turn was positively related to average weekly alcohol consumption. Frone and colleagues (1994) tested the mediating role of both role-related and general negative emotions. They reported that work-family conflict was positively related to both job and family dissatisfaction, which were positively related to general psychological distress (i.e., combined symptoms of depression and anxiety) that was in turn positively related to heavy drinking.

### Moderation Model

Only one study tested the moderation model. Frone and colleagues (1993) tested the moderating role of tension-reduction expectancies, which are the belief that alcohol promotes relaxation and alleviates negative emotions. They reported that work-family conflict was positively related to drinking to cope and problem drinking only among people with strong tension-reduction expectancies.

## Conclusions

Research on work stress (work stressors and work-family conflict) and alcohol use is growing—the number of studies published on the subject grew from 17 in the 1980s (Cooper et al. 1990) to 39 in the 1990s (at the time this article was written). Several conclusions can be drawn from the recent research on work stress and alcohol use. First, research has expanded to include sources of stress within the work role (i.e., work stressors) as well as sources of stress representing the integration of work and family roles (i.e., work-family conflict). Second, evidence is growing that work stressors and work-family conflict are related to alcohol use. Finally, despite a continuing overreliance on the simple cause-effect model, a clear trend exists toward the development and testing of more sophisticated models of work stress and alcohol use. Few studies published during the 1980s moved beyond the simple cause-effect model; however, increasingly sophisticated models have provided insight on how work stressors and work-family conflict are related to alcohol use. These models have also offered a richer picture of the people most at risk for engaging in work stress-induced drinking.

## Future Research

Although research on work stress and alcohol use is increasingly sophisticated, future research could benefit from several refinements (see textbox, p. 290). More attention needs to be devoted to the assessment of work stressors. In the 31 studies reviewed for this article, the most common work stressors studied were job demands, job control, and job complexity. The research evidence suggests that these stressors are related to alcohol use, but we do not know whether they are the most important work stres-sors. Thus, future research should be more systematic and inclusive in its assessment of work stressors. In addition, researchers often develop their own measures of work stressors, even though validated measures exist in the organizational behavior and occupational health literatures. Consequently, the comparability of studies is limited. This problem is partly remedied by Hurrell and colleagues’ (1998) review describing work-stressor measures that could be helpful for future research. Because most research on work stress and alcohol use has used self-report measures of perceived stressors, more attention should be paid to developing and using objective measures of work stressors (Greiner et al. 1997). For example, rather than relying on employee self-reports of whether the work environment is too noisy or the air quality is low, physical measurements of noise and air quality could be used. Likewise, trained observers might rate specific variables, such as workload or conflicts with customers. Examination of general models of work stress, including models of work-family conflict, developed outside the alcohol literature may provide additional insights for alcohol researchers (Frone et al. 1997*c*; Hurrell et al. 1998).

**Issues for Future Research on Work Stress and Alcohol Use**

Future research on work stress and alcohol use should include the following:

- More attention to types of job stressors examined and their measurement
- More attention to types of alcohol use outcomes, including
  —*Level of involvement* (i.e., drinking motives, usual consumption, heavy consumption, and alcohol problems or dependence)
  —*Context of use* (i.e., overall consumption and on-the-job consumption)
- Broader focus on mediators and moderators of the relation between work-related stressors and alcohol use
- Examination of developmental stages, especially the study of employed adolescents and young adults
- Longitudinal designs with closely spaced waves of measurement, such as daily diary studies.

Studies vary widely in the types of alcohol outcomes they assess. One issue is whether the type of alcohol outcome used affects the strength of the relation of work stressors and work-family conflict to alcohol use. Perhaps work stressors are more strongly related to increases in episodes of heavy drinking than they are to increases in average daily consumption. Such differences may explain some of the inconsistencies across studies. Another issue is that little attention has been paid to the context of alcohol use. Most studies use measures of overall alcohol use and have given almost no attention to on-the-job alcohol use. An interesting question is whether different relations exist between work stres-sors and measures of general versus on-the-job alcohol use.

The results summarized in this article demonstrate that the relation between work stress and alcohol consumption is more complex than implied by the simple cause-effect model. Therefore, more attention should be devoted to identifying and testing plausible mediating and moderating variables. Of the four models presented, the moderated mediation model may have the most potential for helping researchers understand the relation between work stress and alcohol use, because it simultaneously addresses the two fundamental issues of *why* and *when* work stressors are related to alcohol use.

In addition, future research should focus on how different developmental stages might play a role in the connection between work stressors and alcohol consumption. For example, the relation between work stressors and alcohol use may be more pronounced among adolescents and young adults because they are just entering the work-force and are the most likely to engage in heavy alcohol use. Extensive literature documents that the number of hours worked per week is cross-sectionally and longitudinally related to higher levels of alcohol use among employed adolescents (for a review, see Frone 1999). This finding suggests that employment has a causal influence on adolescent drinking. Because of the narrow focus on work hours, however, we do not know what it is about the work environment that promotes increases in adolescents’ alcohol use. It could be exposure to work stressors, low social control, or the social and physical availability of alcohol. Frone and Windle (1997) provided initial evidence of the possible role of work stress. They found that job dissatisfaction was positively related to the frequency of drinking and the quantity consumed per drinking occasion in a sample of employed high school students.

The final issue for future research is the need for longitudinal studies of work stress and alcohol use. Crum and colleagues (1995) found that workload and job control predicted new cases of alcohol abuse and alcohol dependence over a 12-month period, and Frone and colleagues (1997*b*) found that work-family conflict predicted increases in heavy drinking over a 4-year period. Nonetheless, scant longitudinal data exist in the literature. Although we can conclude that work stressors and work-family conflict are related to alcohol use, the causal direction of this relation is still unclear because of the heavy reliance on cross-sectional research designs. In future longitudinal research, daily or weekly diary studies (in which participants record their drinking behaviors and stressors each day) would be especially useful. Because variations in exposure to stressors and drinking behaviors may follow a short-term (daily or weekly) cycle, diary methods are likely to be more sensitive than traditional panel designs, which follow a group of study participants over time but collect data at time points that are separated by several months to several years.

## Footnotes

1 Most studies of work stress have used a broad array of alcohol consumption measures (e.g., drinks per day, frequency of drinking, drinks per drinking occasion, and frequency of heavy drinking) or nondiagnostic measures of problematic alcohol use (e.g., a count of the number of alcohol-related problems experienced). For general discussion, the term “alcohol use” is used to refer collectively to all possible alcohol outcomes. When reviewing a specific study, alcohol consumption is described as it was assessed (e.g., average monthly consumption). If a study used an outcome variable that reflected general and nondiagnostic alcohol-related problems, it is referred to as problem drinking. The terms “alcohol abuse” and “alcohol dependence” are only used when the study being reviewed used criteria defined in the American Psychiatric Association’s *Diagnostic and Statistical Manual of Mental Disorders* (DSM–III, DSM–III–R, and DSM–IV).

2 For this article, the author located a total of 31 studies published during the 1990s that examined the relation between work stressors and alcohol use. A complete list of those studies can be obtained from the author. All studies that specifically examined the relation of work-family conflict to alcohol use are cited in this article.

3 The definition of the term “heavy drinking” can vary from study to study. However, researchers usually measure “heavy drinking” based on the frequency in which a subject consumes five or more drinks per day or per sitting or the frequency of drinking to intoxication. A standard drink is defined as one 12-ounce beer, one 5-ounce glass of wine, or 1.5 ounces of distilled spirits.

4 Identity theory suggests that people define who they are in terms of social roles (e.g., employee, spouse, and parent). However, a given social role (e.g., employee) may not be equally important for self-definition for all people. In other words, the psychological importance of social roles may vary across people. Therefore, problems that occur in a given social role (e.g., conflict with one’s supervisor) may or may not be experienced as stressful. For example, work stressors should only have a negative effect on people who define themselves in terms of their work, whereas job stressors should not affect people who do not define themselves in terms of their work.

5 Alcohol expectancies are a drinker’s expectations of how alcohol will affect him or her. For example, people with positive alcohol expectancies may believe that alcohol relaxes them and makes them more socially competent.

## References

[b1-arh-23-4-284] Ames GM, Grube JW (1999). Alcohol availability and workplace drinking: Mixed method analyses. Journal of Studies on Alcohol.

[b2-arh-23-4-284] Ames GM, Janes C (1992). A cultural approach to conceptualizing alcohol and the workplace. Alcohol Health & Research World.

[b3-arh-23-4-284] Ames GM, Grube JW, Moore RS (1997). The relationship of drinking and hangovers to workplace problems: An empirical study. Journal of Studies on Alcohol.

[b4-arh-23-4-284] Bromet EJ, Dew MA, Parkinson DK, Eckenrode J, Gore S (1990). Spillover between work and family: A study of blue-collar working women. Stress Between Work and Family.

[b5-arh-23-4-284] Cooper ML, Russell M, Frone MR (1990). Work stress and alcohol effects: A test of stress-induced drinking. Journal of Health and Social Behavior.

[b6-arh-23-4-284] Crum RM, Muntaner C, Eaton WW, Anthony JC (1995). Occupational stress and the risk of alcohol abuse and dependence. Alcoholism: Clinical and Experimental Research.

[b7-arh-23-4-284] Dawson DA (1994). Heavy drinking and the risk of occupational injury. Accident Analysis and Prevention.

[b8-arh-23-4-284] Frone MR (1998). Predictors of work injuries among employed adolescents. Journal of Applied Psychology.

[b9-arh-23-4-284] Frone MR, Barling J, Kelloway EK (1999). Developmental consequences of youth employment. Young Workers: Varieties of Experience.

[b10-arh-23-4-284] Frone MR Work-family conflict and employee psychiatric disorders: The national comorbidity survey. Journal of Applied Psychology.

[b11-arh-23-4-284] Frone MR, Windle M (1997). Job dissatisfaction and substance use among employed high school students: The moderating influence of active and avoidant coping styles. Substance Use and Misuse.

[b12-arh-23-4-284] Frone MR, Russell M, Cooper ML (1993). Relationship of work-family conflict, gender, and alcohol expectancies to alcohol use/abuse. Journal of Organizational Behavior.

[b13-arh-23-4-284] Frone MR, Barnes GM, Farrell MP (1994). Relationship of work-family conflict to substance use among employed mothers: The role of negative affect. Journal of Marriage and the Family.

[b14-arh-23-4-284] Frone MR, Russell M, Barnes GM (1996). Work-family conflict, gender, and health-related outcomes: A study of employed parents in two community samples. Journal of Occupational Health Psychology.

[b15-arh-23-4-284] Frone MR, Russell MR, Cooper ML (1997a). Job stressors, job involvement, and employee health: A test of identity theory. Journal of Occupational and Organizational Psychology.

[b16-arh-23-4-284] Frone MR, Russell M, Cooper ML (1997b). Relation of work-family conflict to health outcomes: A four-year longitudinal study of employed parents. Journal of Occupational and Organizational Psychology.

[b17-arh-23-4-284] Frone MR, Yardley JK, Markel K (1997c). Developing and testing an integrative model of the work-family interface. Journal of Vocational Behavior.

[b18-arh-23-4-284] Greenberg ES, Grunberg L (1995). Work alienation and problem alcohol behavior. Journal of Health and Social Behavior.

[b19-arh-23-4-284] Greiner BA, Ragland DR, Krause N, Syme SL, Fisher JM (1997). Objective measurement of occupational stress factors: An example with San Francisco urban transit operators. Journal of Occupational Health Psychology.

[b20-arh-23-4-284] Grunberg L, Moore S, Greenberg ES (1998). Work stress and problem alcohol behavior: A test of the spill-over model. Journal of Organizational Behavior.

[b21-arh-23-4-284] Grunberg L, Moore S, Anderson-Connolly R, Greenberg ES (1999). Work stress and self-reported alcohol use: The moderating role of escapist reasons for drinking. Journal of Occupational Health Psychology.

[b22-arh-23-4-284] Hemmingsson T, Lundberg I (1998). Work control, work demands, and work social support in relation to alcoholism among young men. Alcoholism: Clinical and Experimental Research.

[b23-arh-23-4-284] Hurrell JJ, Nelson DL, Simmons BL (1998). Measuring job stressors and strains: Where we have been, where we are, and where we need to go. Journal of Occupational Health Psychology.

[b24-arh-23-4-284] Kawakami N, Araki S, Haratani T, Hemmi T (1993). Relations of work stress to alcohol use and drinking problems in male and female employees of a computer factory. Environmental Research.

[b25-arh-23-4-284] Martin JK, Roman PM (1990). Jobs, occupations, and patterns of alcohol consumption: A review of literature. Alcohol Problem Intervention in the Workplace.

[b26-arh-23-4-284] Martin JK, Kraft JM, Roman PM, Macdonald S, Roman PM (1994). Extent and impact of alcohol and drug use problems in the workplace: A review of empirical evidence. Research Advances in Alcohol and Drug Problems. Volume II: Drug Testing in the Workplace.

[b27-arh-23-4-284] Martin JK, Roman PM, Blum TC (1996). Job stress, drinking networks, and social support at work: A comprehensive model of employees’ problem drinking. Sociological Quarterly.

[b28-arh-23-4-284] Normand J, Lempert RO, O’Brien CP (1994). Under the Influence? Drugs and the American Workforce.

[b29-arh-23-4-284] Parker DA, Farmer GC, Roman PM (1990). Employed adults at risk for diminished self-control over alcohol use: The alienated, the burned out, and the unchallenged. Alcohol Problem Intervention in the Workplace.

[b30-arh-23-4-284] Parker DA, Harford TC (1992). Gender-role attitudes, job competition and alcohol consumption among women and men. Alcoholism: Clinical and Experimental Research.

[b31-arh-23-4-284] Ragland DR, Greiner BA, Krause N, Holman BL, Fisher JM (1995). Occupational and nonoccu-pational correlates of alcohol consumption in urban transit drivers. Preventive Medicine.

[b32-arh-23-4-284] Roman PM, Blum TC, Coombs RH, Ziedonis DM (1995). Systems-oriented prevention strategies and programs: Employers. Handbook on Drug Abuse Prevention: A Comprehensive Strategy to Prevent the Abuse of Alcohol and Other Drugs.

[b33-arh-23-4-284] Romelsjo A, Hasin D, Hilton M, Bostrom G, Diderichsen F, Haglund B, Hallqvist J, Karlsson G, Svanstrom L (1992). The relationship between stressful working conditions and high alcohol consumption and severe alcohol problems in an urban general population. British Journal of Addiction.

[b34-arh-23-4-284] Roxburgh S (1998). Gender differences in the effect of job stressors on alcohol consumption. Addictive Behaviors.

[b35-arh-23-4-284] Shore ER (1992). Drinking patterns and problems among women in paid employment. Alcohol Health & Research World.

[b36-arh-23-4-284] Steptoe A, Wardle J, Lipsey Z, Mills R, Oliver G, Jarvis M, Kirschbaum C (1998). A longitudinal study of workload and variations in psychological well-being, cortisol, smoking, and alcohol consumption. Annals of Behavioral Medicine.

[b37-arh-23-4-284] Trice HM, Sonnenstuhl WJ (1990). On the construction of drinking norms in work organizations. Journal of Studies on Alcohol.

[b38-arh-23-4-284] Vasse RM, Nijhuis FJN, Kok G (1998). Associations between work stress, alcohol consumption, and sickness absence. Addiction.

[b39-arh-23-4-284] Wilsnack RW, Wilsnack SC (1992). Women, work, and alcohol: Failures of simple theories. Alcoholism: Clinical and Experimental Research.

